# Sex differences in the immune response to acute COVID-19 respiratory tract infection

**DOI:** 10.1186/s13293-021-00410-2

**Published:** 2021-12-20

**Authors:** Shaohua Qi, Conelius Ngwa, Diego A. Morales Scheihing, Abdullah Al Mamun, Hilda W. Ahnstedt, Carson E. Finger, Gabriela Delevati Colpo, Romana Sharmeen, Youngran Kim, HuiMahn A. Choi, Louise D. McCullough, Fudong Liu

**Affiliations:** 1 Department of Neurology, McGovern Medical School, The University of Texas Health Science Center at Houston, 6431 Fannin Street, Houston, TX 77030 USA; 2 Department of Neurosurgery, McGovern Medical School, The University of Texas Health Science Center at Houston, 6431 Fannin Street, Houston, TX 77030 USA

**Keywords:** COVID-19, Cytokine, Leukocytes, Immune response, Sex difference

## Abstract

**Background:**

Sex differences in COVID-19 are increasingly recognized globally. Although infection rates are similar between the sexes, men have more severe illness. The mechanism underlying these sex differences is unknown, but a differential immune response to COVID-19 has been implicated in several recent studies. However, how sex differences shape the immune response to COVID-19 remains understudied.

**Methods:**

We collected demographics and blood samples from over 600 hospitalized patients diagnosed with COVID-19 from May 24th 2020 to April 28th, 2021. These patients were divided into two cohorts: Cohort 1 was further classified into three groups based on the severity of the disease (mild, moderate and severe); Cohort 2 patients were longitudinally followed at three time points from hospital admission (1 day, 7 days, and 14 days). MultiPlex and conventional ELISA were used to examine inflammatory mediator levels in the plasma in both cohorts. Flow cytometry was conducted to examine leukocyte responses in Cohort 2.

**Results:**

There were more COVID^+^ males in the total cohort, and the mortality rate was higher in males vs. females. More male patients were seen in most age groups (in 10-year increments), and in most ethnic groups. Males with severe disease had significantly higher levels of pro-inflammatory cytokines (IL-6, IL-8, MCP-1) than females; levels of IL-8, GRO, sCD40L, MIP-1β, MCP-1 were also significantly higher in severe vs. mild or control patients in males but not in females. Females had significantly higher anti-inflammatory cytokine IL-10 levels at 14 days compared to males, and the level of IL-10 significantly increased in moderate vs. the control group in females but not in males. At 7 days and 14 days, males had significantly more circulating neutrophils and monocytes than females; however, B cell numbers were significantly higher in females vs. males.

**Conclusion:**

Sex differences exist in hospitalized patients with acute COVID-19 respiratory tract infection. Exacerbated inflammatory responses were seen in male vs. female patients, even when matched for disease severity. Males appear to have a more robust innate immune response, and females mount a stronger adaptive immune response to COVID-19 respiratory tract infection.

**Supplementary Information:**

The online version contains supplementary material available at 10.1186/s13293-021-00410-2.

## Background

Coronavirus Disease 2019 (COVID-19) caused by the severe acute respiratory syndrome coronavirus 2 (SARS-CoV-2) has been a worldwide pandemic, with over 32 million people in the US infected as of May 2021 [1]. About 20% of those infected with COVID-19 require hospitalization, of which 5–10% require intensive care therapy. The COVID-19 virus is transmitted through respiratory droplets from coughing and sneezing, and enters the nasal system to replicate and propagate. There has been a lack of efficacious therapies for the virus, and thus the immune system remains the best defense [2]. Once the human body is infected, immune cells are mobilized, and in many cases “cytokine storm” occurs in an effort to combat the virus [3]. It is increasingly recognized that COVID-19 disproportionately affects men compared to women [4, 5]. Preliminary studies have suggested that while the prevalence of infection is the same in men and women, male patients are more likely to be hospitalized, have a more severe course of disease and higher mortality [6]. Sex differences are well known in innate and adaptive immunity with resultant sex-specific responses to vaccines and infections [7], which has been postulated as an important reason for the differential response across sex to COVID-19.

To better understand the profiles of sex differences in immune responses to COVID-19, in this study we examined the plasma inflammatory mediators and peripheral blood immune cells from hospitalized patients (both sexes) with COVID-19, and at multiple time points. We quantified immune cell mobilization not only based on the disease severity, but also longitudinally.

## Methods

### Sampling and cohorts

Samples were collected from consented healthy donors and hospitalized patients with acute COVID-19 respiratory tract infection (at the Memorial Hermann Hospital System) from May 24th 2020 to April 28th, 2021 (see Additional file 1 for the Inclusion/Exclusion Criteria). The average age of these patients was 53.49 ± 16.89, with the majority being between 41 and 65 years old (age ranged from 19 to 101). Males constituted 60.0% of the sample (*N* = 350 male and 276 females). American Indians or Alaska Natives constituted 0.16%, Asians 3.14%, African Americans 21.9%, Hispanics 4.4%, Whites 62.9%, and unable to determine (UTD) 7.5% of patients. All collected samples were divided into two cohorts as a smaller subset of patients were hospitalized for over 2 weeks. Cohort 1 patients were graded for severity based on respiratory symptoms and the need for supplemental oxygen therapy according to the classification from the NIH COVID-19 spectrum [8] and the therapeutic management of COVID-19 [9]: *mild*, nasal cannula less than 5 L and less than 5 days in the hospital; *moderate*, nasal cannula more than 5 L or high-flow oxygen therapy and more than 5 days in the hospital; *severe*, ventilator or died because of COVID-19. Blood samples from Cohort 1 patients were collected within 3–4 days of hospital admission, and Cohort 2 samples were collected on day 1, day 7, and day 14 from admission. Briefly, the blood sample (5 mL) was obtained by venipuncture or from an existing line cleared of I.V. solution (venipuncture, arterial or venous recorded) in 6-mL K2 EDTA Vacutainer tubes (BD, Franklin Lakes, NJ, USA). Samples were processed within one hour of collection. Blood cells were removed by centrifugation (4 °C, 800×*g* for 10 min), and the platelet-poor plasma collected after another centrifugation (4 °C, 10,000×*g* for 10 min) and stored at − 80 °C until all samples were ready for analysis. All assessments were performed blinded to the subject’s history of COVID-19. The study was approved by the Institutional Review Board (IRB) of University of Texas Health Science Center at Houston (HSC-MS-17-0452).

### Multiplex and conventional ELISA

Multiplex enzyme-linked immunosorbent assays were performed on plasma of Cohort 1 for inflammatory mediators including cytokines and chemokines using a commercially available panel (MILLIPLEX® Human Cytokine/Chemokine/Growth Factor Panel A—Immunology Multiplex Assay HCYTA-60K-08, Millipore Sigma) by following the manufacturer’s instructions. The inflammatory mediators in the panel of the kit are: sCD40L, EGF, Eotaxin, FGF-2, Flt-3 ligand, Fractalkine, G-CSF, GM-CSF, GROα, IFNα2, IFNγ, IL-1α, IL-1β, IL-1ra, IL-2, IL-3, IL-4, IL-5, IL-6, IL-7, IL-8, IL-9, IL-10, IL-12 (p40), IL-12 (p70), IL-13, IL-15, IL-17A, IL-17E/IL-25, IL-17F, IL-18, IL-22, IL-27, IP-10, MCP-1, MCP-3, M-CSF, MDC (CCL22), MIG, MIP-1α, MIP-1β, PDGF-AA, PDGF-AB/BB, RANTES, TGFα, TNFα, TNFβ, and VEGF-A (see Additional file 2 for details).

Conventional ELISA was used to examine IL-6, IL-8, IL-10, TNF-α, and TGF-β1 in plasma of Cohort 2 patients at three time points (1 day, 7 days, 14 days) by using human ELISA LEGEND MAX (IL-6_430507, IL-8_431507, IL-10_430607, TNF-α_430207, and TGF-β1_436707; BioLegend). Signals were read at 450 nm in EnSpire™ Multimode Plate Reader (PerkinElmer, Inc.).

### Flow cytometry

Flow cytometry assay was conducted on the blood samples of Cohort 2. Briefly, each human blood aliquot (100 µL) in autoclaved Eppendorf tube was added pre-chilled HBSS containing 2% FBS (1 mL), and then followed by centrifuging at 500×*g* for 5 min and at 4 °C. The pellets (cells) were re-suspended by adding another pre-chilled HBSS containing 2% FBS (200 µL) and blocked in human Fc Block (422302, Biosciences) (1 µL/sample). For live/dead cell discrimination, a fixable viability dye lock, carboxylic acid succinimidyl ester was used (CASE-AF350, Invitrogen). Next, the cells were stained by adding the antibody mix specific for each cell population (Tables 1, 2 and 3) and incubated for 30 min at 4 °C on a rotator in the dark and at 4 °C. Isotype control antibodies were used in parallel with the cell specific antibodies to remove the nonspecific signals in each panel. After antibody incubation, the samples were suspended in pre-chilled HBSS containing 2% FBS (1 mL) and centrifuged at 500×*g* for 5 min and at 4 °C. The pellets (cells) were suspended again and fixed in ice-cold 1% PFA (200 µL) for 10 min and in the dark. Following another wash, red blood cells were lysed by adding 2 mL of 1× RBC Lysis buffer (420302, BioLegend) at room temperature (Thermo Fisher Scientific, Waltham, MA, USA) for 10 min. The samples were washed again and centrifuged at 500×*g* for 5 min and at 4 °C, and then re-suspended in pre-chilled HBSS containing 2% FBS (300 µL), and immediately assayed in a flow cytometer (CytoFLEX b75408, Beckman Coulter). Data were analyzed by using FlowJo_v10.7.2 (Tree Star Inc.).

**Table 1:** Antibody panel for neutrophil flow

Antibody	Source	Catalog no.
BUV395 mouse anti-human CD45 antibody	BD Biosciences	563792
BV421 mouse anti-human CD11b antibody	BD Biosciences	562632
6g8-AF568 anti-DEspR antibody	LakePharma	N/A
LIVE/DEAD™ fixable aqua dead cell stain kit	Thermo Fisher	L-34966

**Table 2:** Antibody panel for lymphocyte flow

Antibody	Source	Catalog no.
BUV395 mouse anti-human CD45 antibody	BD Biosciences	563792
BV421 mouse anti-human CD11b antibody	BD Biosciences	562632
Brilliant Violet 421™ anti-human CD3 antibody	BioLegend	317344
PE anti-human CD19 antibody	BioLegend	392506
LIVE/DEAD™ fixable aqua dead cell stain kit	Thermo Fisher	L-34966

**Table 3:** Antibody panel for monocyte flow

Antibody	Source	Catalog no.
BUV395 mouse anti-human CD45 antibody	BD Biosciences	563792
Brilliant Violet 785™ anti-human CD16 antibody	BioLegend	302046
LIVE/DEAD™ fixable aqua dead cell stain kit	Thermo Fisher	L-34966
APC anti-human CD14 antibody	BioLegend	367118
PE anti-human CD24 antibody	BioLegend	311106

### Statistical analyses

Cytokine and immune cell data were presented as the mean ± standard error of the mean (SEM), and comparisons were made with the severity of COVID-19 and sex using two-way ANOVA plus Sidak’s post hoc correction. Prevalence of other organ involvement or comorbidities, in addition to pneumonia, were presented as % of patients with 95% confidence intervals according to the severity of COVID-19, time point since admission, and sex. Statistical differences between the sexes were determined using Chi-square tests and any trends across the levels of severity or follow-up time points were assessed using the extended Mantel–Haenszel Chi-square for linear trend. All statistical data analysis was performed using Graph-Pad PRISM Version 9.0 (GraphPad Software, CA, USA) and statistically significance levels were set at *p* < 0.05 for 2-tailed tests.

## Results

### Male sex is a risk factor for acute COVID-19 respiratory tract infection

**Fig. 1:**
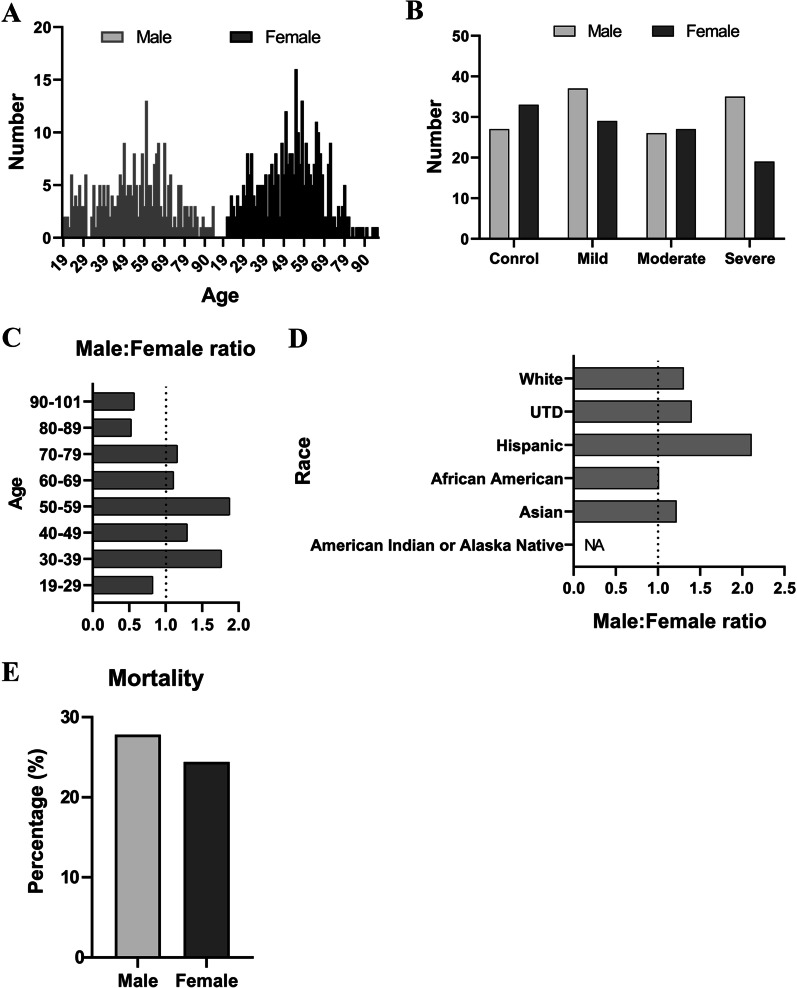
Male sex is a risk factor for acute COVID-19 respiratory tract infection. **A** Age distribution of COVID-19 patients. **B** Male and female patients based on clinical classification of severity of COVID-19. **C** Male-to-female ratios of COVID-19 cases in 10 years of age increments. **D** Male-to-female ratios of COVID-19 cases based on ethnicity. **E** Mortality rate in males vs. females

A sex imbalance in the hospitalization and mortality from COVID-19 patients has been reported worldwide [10–14]. We examined the sex (self-identified) distribution in our hospitalized patients that were graded into three groups (mild, moderate and severe) [8]. There were more males than females in the mild and severe group (Fig. 1A, B). We also analyzed the male/female ratio by age in 10-year increments. In most age groups, the ratios were above one, indicating more male vs. female patients (Fig. 1C). Interestingly, when analyzed based on ethnicity, the male/female ratios were above one in almost all ethnicities except for African Americans (Fig. 1D). The overall mortality rate was higher in male vs. female patients (Fig. 1E).

### Inflammatory mediator levels in plasma after acute COVID-19 respiratory tract infection

**Fig. 2:**
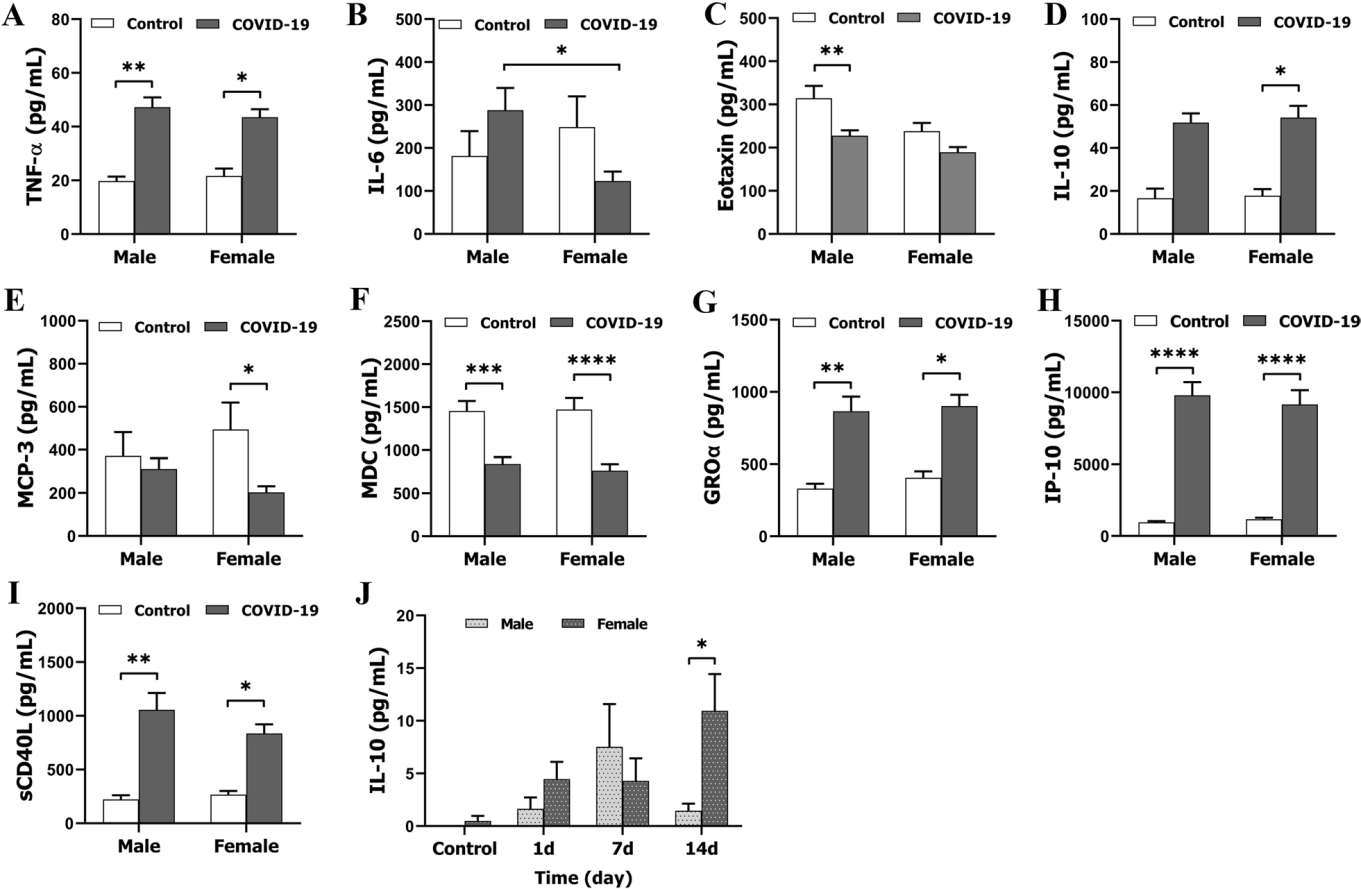
Levels of inflammatory mediators in control vs. COVID-19 plasma samples. **A**–**I** Levels of TNF-α, IL-6, Eotaxin, IL-10, MCP-3, MDC (CCL22), GROα, IP-10, and sCD40L, respectively, in Cohort 1 patients measured by MultiPlex ELISA. **J** Levels of anti-inflammatory IL-10 cytokine in Cohort 2 patients by conventional ELISA. *N* = 6–88 independent experiments assayed in duplicates. *p < 0.05; **p < 0.01 ***p < 0.001, ****p < 0.0001; two-way ANOVA with Sidak’s post hoc correction

Previous studies suggested that the severity of COVID-19 is associated with an increased level of inflammatory mediators including cytokines and chemokines [15, 16]. Here, we examined 48 mediators (Additional file 2) in Cohort 1 patients, among which 38 were detectable by our Multiplex ELISA assay. We presented the data of TNF-α, IL-6, IL-8, Eotaxin, IL-10, MCP-3, MDC (CCL22), GROα, IP-10, sCD40L, MIP-1β, and Fractalkine only, as differences between groups were found. However, data of other mediators (EGF, FGF-2, Flt-3 ligand, G-CSF, GM-CSF, IFNα2, IFNγ, IL-1α, IL-1β, IL-1ra, IL-2, IL-3, IL-4, IL-5, IL-7, IL-9, IL-12 (p40), IL-12 (p70), IL-13, IL-15, IL-17A, IL-17E/IL-25, IL-17F, IL-18, IL-22, IL-27, MCP-1, M-CSF, MIG, MIP-1α, PDGF-AA, PDGF-AB/BB, RANTES, TGFα, TNFβ, and VEGF-A) showed no group differences (data not shown). Overall (no disease severity grouping; Fig. 2A–J), IL-6 levels were significantly higher in men vs. women in the COVID groups (Fig. 2B). When compared with controls, Eotaxin in men, and MCP-3 in women were significantly lower (Fig. 2C, E), and IL-10 in women was higher (Fig. 2D), a control vs. COVID-19 difference that was not seen in the counterpart sex, respectively. No sex differences were found in levels of MDC (CCL22), GROα, IP-10, and sCD40L, although males and females exhibited synchronous changes compared to controls (Fig. 2F–I). For samples from Cohort 2 patients, three pro- (IL-6, IL-8, TNF-α) and three anti-inflammatory mediators (IL-10, TGF-β1, CD200) were assayed by conventional ELISA. Sex differences were only seen in IL-10 levels, which were significantly higher in females vs. males at 14 days (Fig. 2J). Together with the data from the cohort 1 patients (Fig. 2D), this suggests that a more robust anti-inflammatory response was seen in female vs. male COVID-19 patients.

### Sex differences in the levels of inflammatory markers are dependent on the severity of COVID-19 illness

**Fig. 3:**
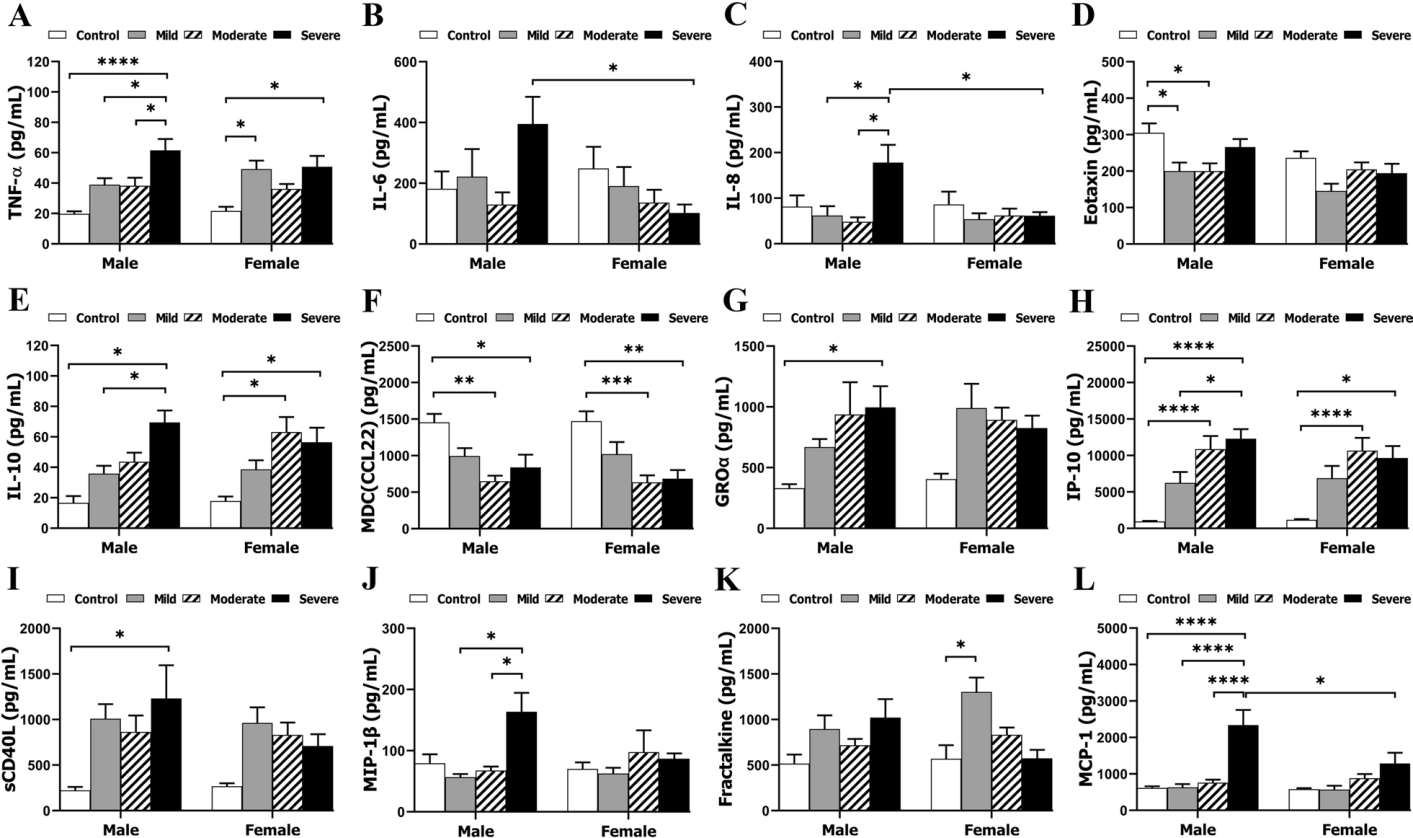
Levels of inflammatory mediators in control vs. COVID-19 plasma samples based on disease severity. **A**–**L** levels of TNF-α, IL-6, IL-8, Eotaxin, IL-10, MDC (CCL22), GROα, IP-10, sCD40L, MIP-1β, Fractalkine, and MCP-1, respectively, in Cohort 1 patients by MultiPlex ELISA. *N* = 6–33 independent experiments assayed in duplicates. *p < 0.05; **p < 0.01 ***p < 0.001, ****p < 0.0001; two-way ANOVA with Sidak’s post hoc correction

The symptoms of COVID-19 are variable, ranging from mild fever to severe illness and intensive care unit (ICU) admission. The severity of the disease may be responsible for the cytokine profiles. We examined if the sex differences seen in the above overall data (Fig. 2) remained in patients with different disease severity (mild, moderate and severe). Interestingly, we found that the most dramatic sex differences were seen in the severe groups, e.g., males in the severe group had significantly higher levels of IL-6, IL-8, and MCP-1 than females (Fig. 3B, C, L), a sex difference not seen in mild or moderate group. Males with severe disease also had significantly higher levels of GROα, sCD40L, and MIP-1β than control, mild, or moderate male groups, which was not seen in females (Fig. 3G, I, J). Of note, compared to controls, the anti-inflammatory cytokine, IL-10, significantly increased in females with moderate and severe disease, but was not significantly elevated until the severe stage in males (Fig. 3E). For TNF-α, Eotaxin, MDC (CCL22), IP-10, and Fractalkine, the data revealed no sex differences. Taken together, male COVID-19 patients with severe illness have an exacerbated immune response compared to females with severe disease.

### Sex differences in circulating immune cells

**Fig. 4:**
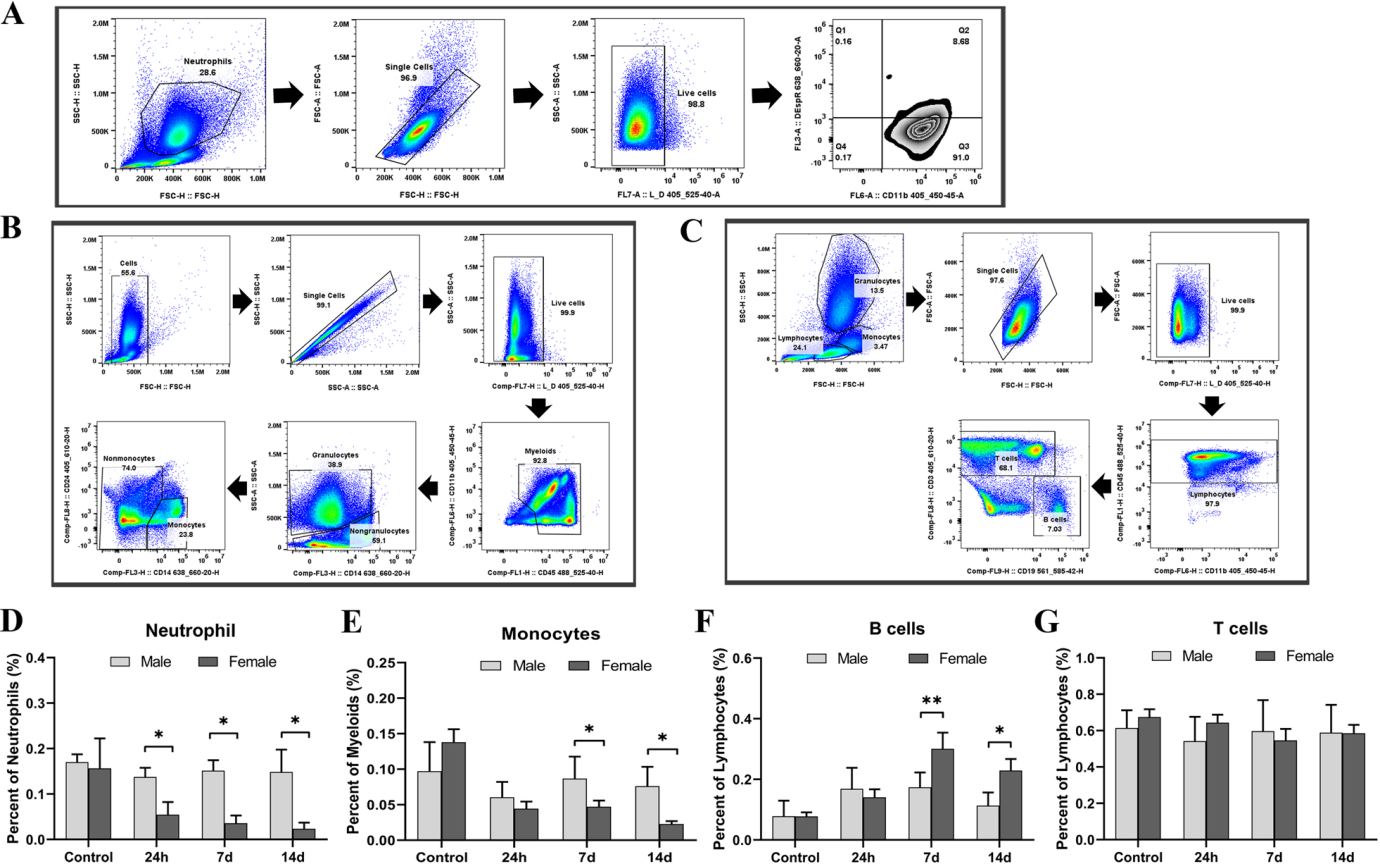
Sex differences in circulating immune cells in Cohort 2 patients at different days of hospitalization days. **A**–**C** Flow cytometric gating strategy for neutrophils, monocytes and lymphocytes in COVID-19 patient peripheral blood. Neutrophils were gated as CD11b^+^DEspR^+^ (**A**), monocytes as CD11b^+^CD45^+^CD14^+^CD24^−^ (**B**), T cells as CD45^+^CD3^+^ and B cells as CD45^+^CD19^+^ (**C**). **D** Percentage of CD11b^+^DEspR^+^ neutrophils in total live leukocytes. **E** Percentage of CD11b^+^CD45^+^CD14^+^CD24^−^ monocytes in total non-granulocytes. **F** Percentage of CD45^+^CD19^+^ B cells in total lymphocytes. **G** Percentage of CD45^+^CD3^+^ T cells in total lymphocytes. *N* = 7–26 independent experiments assayed in duplicates. *p < 0.05, **p < 0.01; two-way ANOVA with Sidak’s post hoc correction

To further evaluate the immune response to COVID-19, we performed flow cytometry on fresh blood samples to examine leukocytes from Cohort 2 patients at three time points (24 h, 7 days, and 14 days) since hospital admission. A panel of antibodies to detect neutrophils (Table 1), monocytes (Table 2), and lymphocytic T and B cells (Table 3) was used to identify these leukocyte subsets. Active neutrophils were gated as CD11b^+^DEspR^+^ [17–19] (Fig. 4A), monocytes as CD11b^+^CD45^+^CD14^+^CD24^−^ (Fig. 4B), T cells as CD45^+^CD3^+^ and B cells as CD45^+^CD19^+^ (Fig. 4C). Interestingly, sex differences were found in both myeloid cells (neutrophils/monocytes) and lymphocytes, but exhibited different patterns by sex. Male patients had more neutrophils and monocytes at each time point (all significant except a trend at 24 h for monocytes) than female patients (Fig. 4D, E). However, there was a significant elevation in B cells in females vs. males at both 7 days and 14 days (Fig. 4F). No sex difference was seen in T cells (Fig. 4G).

### Pneumonia and other organ involvement

**Fig. 5:**
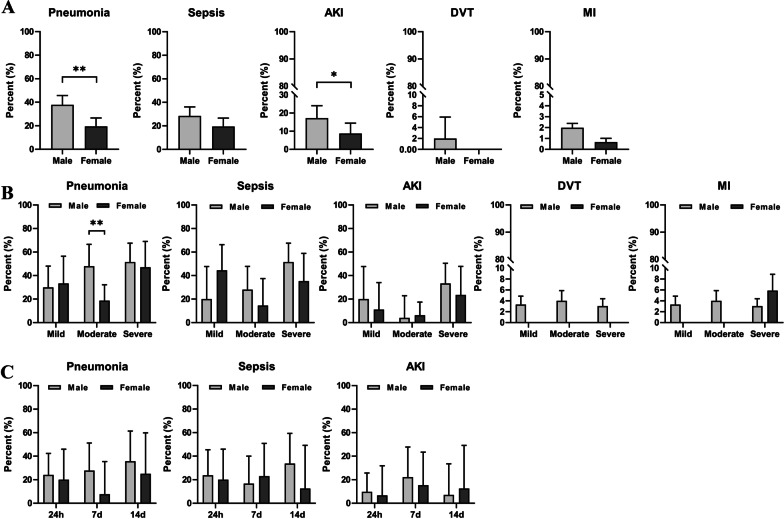
Pneumonia and other organ involvement. **A** Case percentages of pneumonia, sepsis, AKI, DVT and myocardial infarction in all male and female patients. **B**, **C** Case percentages broken down from (**A**) for Cohort 1 (**B**) and for Cohort 2 (**C**) patients. *N* = 8–300 cases/group. Error bars denote upper 95% confidence interval. *p < 0.05, **p < 0.01; Chi-square tests

After infection with COVID-19, patients often have signs of other organ involvement or comorbidities in addition to pneumonia. We reviewed the hospitalization history of our patients and analyzed organ involvement. Figure 5A shows the percentage of pneumonia, sepsis, acute kidney injury (AKI), deep vein thrombosis (DVT), and myocardial infarction in all patients. Overall, more male patients develop these complications than females, with significant differences in pneumonia and AKI. Figure 5B and C shows the percentages of these diseases in Cohort 1 and Cohort 2 patients, respectively. In males with moderate disease, the percentage of pneumonia was significantly higher than that of females. Prevalence of sepsis showed an increasing trend across the severity of COVID-19 infection in males (*p* = 0.012) but not in females.

## Discussion

The immune response to viral infection plays an important role in tissue damage caused by COVID-19 [20]. In the present study, we analyzed the immune response in patients with acute COVID-19 respiratory tract infection both at the cytokine level and the cellular level, and found several novel findings. Firstly, in patients hospitalized with COVID-19, as seen by others, men were overrepresented, and overall had more robust inflammation than females, a sex difference that was particularly pronounced in patients with severe disease. Secondly, although female patients also showed a pro-inflammatory response, they appear to mount an anti-inflammatory response earlier than male patients. Thirdly, our data suggest that early innate immune responses to COVID-19 are more pronounced in men, and delayed adaptive immunity is more highly activated in women. To increase the accuracy and precision in our data interpretation, we analyzed the immune response longitudinally and also disaggregated the data based on the severity of the disease; therefore, we believe our data are convincing and reflect the nature of the immune response in acute COVID-19 respiratory tract infection patients.

Previous clinical data have shown that although the prevalence of infection is the same in men and women, men are more likely to be hospitalized, have a more severe disease course, and higher mortality than women [21]. Our findings are consistent with these reports, as male patients outnumbered females not only in total numbers (350 vs. 276), but also in most age and ethnic groups, and males had higher mortality than females (Fig. 1). In addition, more male patients developed pneumonia, sepsis, AKI, DVT and myocardial infarction than females after COVID-19 infection. A number of reasons have been postulated for the sex differences in the response to COVID-19. Some studies have shown that men have a higher level of ACE2 receptors, used for viral entry into target cells. The cellular serine protease TMPRSS2, responsible for coronavirus spike (S) protein priming, is also highly expressed in the prostate epithelium and is sensitive to androgens [22]. Differences in hormone levels may also underlie the observed sex differences [11]. The present study provides evidence that in addition to the above, the differential immune responses seen in male vs. female patients may also play an important role in mediating the sex differences seen in COVID-19 patients.

Previous studies have found that more severe clinical symptoms of COVID-19 were associated with elevated levels of inflammatory markers [23] and predict poorer prognosis and mortality [24]. In the present study, circulating levels of inflammatory mediators appear to reflect the systemic inflammation caused by the infection. It is intriguing that sex differences in the cytokine levels were found both in the overall data (Fig. 2) and in the graded groups based on disease severity (Fig. 3). Although the overall data provided limited information as the cytokine levels were calculated regardless of the disease severity, sex differences were evident in the pro-inflammatory cytokine IL-6 (higher in males) and the anti-inflammatory cytokine IL-10 (higher in females). When we analyzed the data in the graded groups (Fig. 3), more sex differences were revealed, especially in the severe group. Of the 12 inflammatory mediators, 6 (IL-6, IL-8, GROα, sCD4L, MIP-1β, MCP-1) exhibited very high levels in males with severe disease but not in females, all of which are pro-inflammatory mediators. The anti-inflammatory cytokine IL-10 again exhibited a female favorable phenotype, i.e., the level significantly increased in the moderate group but not in the males compared to controls. IL-10 is well known for its beneficial roles in inflammation resolution and tissue repair in inflammatory diseases [25]. The higher level of IL-10 in female vs. male patients may be an important reason why female patients have a better prognosis and lower mortality.

Our data also revealed a sex difference in the leukocyte response to acute COVID-19 respiratory tract infection. While the innate immune response seemed to be more active in male patients indicated by higher numbers of circulating inflammatory neutrophils and monocytes, the adaptive immune response was more robust in females, and females had significantly more B cells at 7 days and 14 days (Fig. 4F). B cells possess distinct machinery for adaptive immunity compared to other immune cells. Upon activation, B cells proliferate rapidly and differentiate into antibody-secreting plasma cells; antibodies released from these cells bind specifically to foreign antigens (e.g., viruses) and inactivate viruses and microbial toxins [26]. In addition to antibody secretion, B cells also regulate T cell responses affecting the progression of diseases; therefore, B cell-induced adaptive immunity is both cellular and humoral [27]. On the other hand, neutrophils are a major component of the innate immune response [28] and monocytes/macrophages are members of the mononuclear phagocytic system, are also a component of innate immunity [29]. While the innate immune response is immediate, the effect of the adaptive immune response is long-lasting and highly specific. Another cell type that participates in adaptive immunity is the T cell. Somewhat surprisingly, we did not see any significant sex difference in T cells in our data (Fig. 4G). Although both innate and adaptive immunity are important in the fight against foreign antigens, the present study suggests that the adaptive immunity in female patients is more robust and sustained in females compared to male patients. The chronic consequences of this sex-specific activation of B cells remain unknown.

The present study has some limitations that should be kept in mind when interpreting the data. The age of all recruited patients ranges from 19 to 101, and aged patients may have more complications; however, our control participants were healthy with an age range of 25–60. Therefore, age-matching between controls vs. patients was not ideal. We also did not measure circulating sex hormone levels (estrogen and testosterone) in our patients; therefore, the contribution of the gonadal hormones to the sex difference seen in immunity is not known. We also did not deeply phenotype the immune cells, and contributions of specific lower prevalence cells will require further study. Of note, the difference in immune responses between males vs. females may not be solely related to acute lung injury (pneumonia); instead, it may reflect a systemic response, as males also had higher incidence of other organ involvement than females (Fig. 5).

## Perspectives and significance

In conclusion, the present study examined sex differences in patients with acute COVID-19 respiratory tract infection, with a focus on the immune responses. Examining inflammatory mediators, we found female patients launched a more robust anti-inflammatory response than males. Using flow cytometry to analyze immune cells, the study revealed that innate and adaptive immunity were differentially activated in male vs. female patients. Although these sex differences in the immune response could either be resultant or causative to the COVID-19 virus infection, we conclude that the “female favorable” phenotype of COVID-19 could be at least in part due to the higher levels of anti-inflammatory factors and earlier activation of adaptive immunity.

## Supplementary Information

**Additional file 1.** Inclusion and exclusion criteria.

**Additional file 2.** Inflammatory Mediator list.

## Abbreviations

COVID-19: Coronavirus Disease 2019
SARS-CoV-2: Severe acute respiratory syndrome coronavirus 2
ICU: Intensive Care Unit
DEspR: Dual endothelin-1/VEGF signal peptide receptor
ACE2: Angiotensin converting enzyme 2
TLR7: Toll-like receptor 7
MCP-3: Monocyte-chemotactic protein-3
MDC (CCL22): Macrophage-derived chemokine (CCL22)
GROα: Growth-related oncogene α
IP-10: Interferon gamma-induced protein 10
sCD40L: Soluble form of CD40L
MIP-1β: Macrophage inflammatory protein-1β
MCP-1: Monocyte chemoattractant protein-1
