# Supplementary material for: Sex differences in the immune response to acute COVID-19 respiratory tract infection
Source: Biol Sex Differ. 2021 Dec 20;12:66. doi: 10.1186/s13293-021-00410-2 (PMC8686792; doi:10.1186/s13293-021-00410-2)
Supplement: Supplementary file 1 — Additional file 1. Inclusion and exclusion criteria. [file 13293_2021_410_MOESM1_ESM.docx]

**Additional File 1:**

**Inclusion and exclusion criteria**

| **Inclusion Criteria** | **Exclusion Criteria** |
| --- | --- |
| 1. Laboratory-confirmed infection by real-time PCR  2. Admission to an intensive care unit for COVID-19 and discharge  3. Written informed consent from the patient at the time of discharge from the ICU  4. Outpatients aged ≥ 18 years of age | 1. Patients with severe functional disabilities before hospital admission for COVID-19  2. History of pulmonary complications (including resection and transplant)  3. Pre-existing systemic diseases which would impact outcomes (i.e. stroke, myocardial infarction, pulmonary disease requiring home oxygen, chronic renal failure necessitating hemodialysis, malignancy)  4. Use of inhaled or systemic glucocorticoids within the past 7 days  5. Known allergy or contraindication to budesonide  6. Documented neurologic and psychiatric disorders  7. Prisoners and pregnant women |
