# Supplementary material for: Sex differences in the immune response to acute COVID-19 respiratory tract infection
Source: Biol Sex Differ. 2021 Dec 20;12:66. doi: 10.1186/s13293-021-00410-2 (PMC8686792; doi:10.1186/s13293-021-00410-2)
Supplement: Supplementary file 2 — Additional file 2. Inflammatory Mediator list. [file 13293_2021_410_MOESM2_ESM.docx]

**Inflammatory Mediator list**

| **Abbr.** | **Full name** | **Pro- or anti-inflammatory** | **COVID-19 related characteristics** |
| --- | --- | --- | --- |
| sCD40L | soluble form of CD40L | Pro | sCD40L was significantly higher in COVID-19 patients [1, 2] |
| EGF | Epidermal growth factor | Anti |  |
| Eotaxin |  | Pro |  |
| FGF-2 | Fibroblast growth factor-2 | Anti |  |
| Flt-3 ligand | Fms-like tyrosine kinase-3 ligand | Pleiotropic |  |
| Fractalkine | Fractalkine | Anti | Higher levels detected in mildly symptomatic and severe cases compared to asymptomatic [3] |
| G-CSF | Granulocyte colony stimulating factor | Anti |  |
| GM-CSF | Granulocyte-macrophage colony-stimulating factor | Pro |  |
| GROα | growth-related oncogene α | Pro | Significantly increased in patients with severe COVID-19 infection [4] |
| IFNα2 | Interferon α 2 | Pro |  |
| IFNγ | Interferon γ | Pro |  |
| IL-1α | Interleukin-1 α | Pro |  |
| IL-1β | Interleukin-1 β | Pro |  |
| IL-1ra | Interleukin -1 receptor antagonist | Pro |  |
| IL-2 | Interleukin-2 | Anti |  |
| IL-3 | Interleukin-3 | Pro |  |
| IL-4 | Interleukin-4 | Anti |  |
| IL-5 | Interleukin-5 | Pro |  |
| IL-6 | Interleukin-6 | Pro | IL-6 levels were higher in severe COVID-19 patients [5-8] |
| IL-7 | Interleukin-7 | Pro |  |
| IL-8 | Interleukin-8 | Pro | IL-8 plasma levels were elevated in both mild and severe COVID-19 patients [8, 9] |
| IL-9 | Interleukin-9 | Anti |  |
| IL-10 | Interleukin-10 | Anti | IL-10 levels were higher in severe patients [5, 10] |
| IL-12 (p40) | Interleukin-12(subunit p40) | Anti |  |
| IL-12 (p70) | Interleukin-12(subunit p70) | Anti |  |
| IL-13 | Interleukin-13 | Anti |  |
| IL-15 | Interleukin-15 | Anti |  |
| IL-17A | Interleukin-17A | Pro |  |
| IL-17E/IL-25 | Interleukin-17E | Pro |  |
| IL-17F | Interleukin-17F | Pro |  |
| IL-18 | Interleukin-18 | Pro |  |
| IL-22 | Interleukin-22 | Anti |  |
| IL-27 | Interleukin-27 | Anti |  |
| IP-10 | Interferon gamma-induced protein-10 | Pro | Expression levels of IP-10, MCP-3, HGF, MIG, MIP-1α and IL-1ra were highly associated with disease severity and progression [11, 12] |
| MCP-1 | Monocyte chemoattractant protein-1 | Pro |  |
| MCP-3 | Monocyte-chemotactic protein-3 | Pro | MCP-3 were significantly increased in ICU (COVID-19) patients [11, 13] |
| M-CSF | Macrophage colony-stimulating factor | Anti |  |
| MDC (CCL22) | Macrophage-derived chemokine (CCL22) | Pro | CCL22 (also known as MDC) were reduced in COVID-19 patients  relative to HV [14] |
| MIG | Monokine induced by gamma interferon | Pro |  |
| MIP-1α | Macrophage inflammatory protein 1α | Pro |  |
| MIP-1β | Macrophage inflammatory protein-1β | Pro | MIP-1β was higher in patients with severe COVID-19 [15] |
| PDGF-AA | Platelet-derived growth factor (two A subunits) | Anti |  |
| PDGF-AB/BB | Platelet-derived growth factor (one of each or two B subunits) | Anti |  |
| RANTES | Regulated on activation, normal T cell expressed and secreted | Pro |  |
| TGFα | Transforming growth factor α | Anti |  |
| TNFα | Tumor necrosis factor α | Pro | Serum TNF-α levels were greater in ICU and non-ICU patients than in the healthy subjects group [16, 17] |
| TNFβ | Tumor necrosis factor β | Pro |  |
| VEGF-A | Vascular endothelial growth factor | Pleiotropic |  |

1. Campo, G., et al., *Over time relationship between platelet reactivity, myocardial injury and mortality in patients with SARS-CoV-2-associated respiratory failure.* Platelets, 2021. **32**(4): p. 560-567.

2. Patterson, B.K., et al., *Immune-Based Prediction of COVID-19 Severity and Chronicity Decoded Using Machine Learning.* Frontiers in Immunology, 2021. **12**(2520).

3. Khalil, B.A., N.M. Elemam, and A.A. Maghazachi, *Chemokines and chemokine receptors during COVID-19 infection.* Computational and Structural Biotechnology Journal, 2021. **19**: p. 976-988.

4. Zaid, Y., et al., *Platelets Can Associate with SARS-Cov-2 RNA and Are Hyperactivated in COVID-19.* Circ Res, 2020. **127**(11): p. 1404-18.

5. Wan, S., et al., *Characteristics of lymphocyte subsets and cytokines in peripheral blood of 123 hospitalized patients with 2019 novel coronavirus pneumonia (NCP).* medRxiv, 2020: p. 2020.02.10.20021832.

6. Liu, F., et al., *Prognostic value of interleukin-6, C-reactive protein, and procalcitonin in patients with COVID-19.* Journal of clinical virology, 2020. **127**: p. 104370.

7. Han, H., et al., *Profiling serum cytokines in COVID-19 patients reveals IL-6 and IL-10 are disease severity predictors.* Emerging microbes & infections, 2020. **9**(1): p. 1123-1130.

8. Li, L., et al., *Interleukin-8 as a Biomarker for Disease Prognosis of Coronavirus Disease-2019 Patients.* Front Immunol, 2020. **11**: p. 602395.

9. Del Valle, D.M., et al., *An inflammatory cytokine signature predicts COVID-19 severity and survival.* Nature Medicine, 2020. **26**(10): p. 1636-1643.

10. Lu, L., et al., *A Potential Role of Interleukin 10 in COVID-19 Pathogenesis.* Trends Immunol, 2021. **42**(1): p. 3-5.

11. Yang, Y., et al., *Plasma IP-10 and MCP-3 levels are highly associated with disease severity and predict the progression of COVID-19.* J Allergy Clin Immunol, 2020. **146**(1): p. 119-127.e4.

12. Lev, S., et al., *Real-time IP-10 measurements as a new tool for inflammation regulation within a clinical decision support protocol for managing severe COVID-19 patients.* medRxiv, 2020: p. 2020.07.21.20158782.

13. Chen, L., et al., *Scoring cytokine storm by the levels of MCP-3 and IL-8 accurately distinguished COVID-19 patients with high mortality.* Signal Transduction and Targeted Therapy, 2020. **5**(1): p. 292.

14. Abers, M.S., et al., *An immune-based biomarker signature is associated with mortality in COVID-19 patients.* JCI Insight, 2021. **6**(1).

15. Sabioni, L., et al., *Systemic microvascular endothelial dysfunction and disease severity in COVID-19 patients: Evaluation by laser Doppler perfusion monitoring and cytokine/chemokine analysis.* Microvasc Res, 2021. **134**: p. 104119.

16. Mortaz, E., et al., *Increased Serum Levels of Soluble TNF-α Receptor Is Associated With ICU Mortality in COVID-19 Patients.* Front Immunol, 2021. **12**: p. 592727.

17. Feldmann, M., et al., *Trials of anti-tumour necrosis factor therapy for COVID-19 are urgently needed.* The Lancet, 2020. **395**(10234): p. 1407-1409.
